# Association between computed tomography perfusion and the effect of intravenous alteplase prior to endovascular treatment in acute ischemic stroke

**DOI:** 10.1007/s00234-023-03139-4

**Published:** 2023-03-08

**Authors:** Jan W. Hoving, Henk van Voorst, Daan Peerlings, Jasper D. Daems, Miou S. Koopman, Anke Wouters, Manon Kappelhof, Natalie E. LeCouffe, Kilian M. Treurniet, Agnetha A. E. Bruggeman, Leon A. Rinkel, Ido R. van den Wijngaard, Jonathan M. Coutinho, Aad van der Lugt, Henk A. Marquering, Yvo B. W. E. M. Roos, Charles B. L. M. Majoie, Bart J. Emmer, on behalf of the MR CLEAN-NO IV Investigators

**Affiliations:** 1 Department of Radiology and Nuclear Medicine, Amsterdam UMC, location University of Amsterdam, Amsterdam, the Netherlands; 2 Department of Radiology and Nuclear Medicine, Amsterdam University Medical Centers, location University of Amsterdam, Office G1-229, Meibergdreef 9, 1105 AZ Amsterdam, the Netherlands; 3 Department of Biomedical Engineering and Physics, Amsterdam UMC, location University of Amsterdam, Amsterdam, the Netherlands; 4 Department of Radiology, University Medical Center Utrecht, Utrecht, the Netherlands; 5 Department of Public Health, Erasmus University Medical Center, Rotterdam, the Netherlands; 6 Department of Neurology, Erasmus University Medical Center, Rotterdam, the Netherlands; 7 Department of Neurology, Amsterdam University Medical Centers, location University of Amsterdam, Amsterdam, the Netherlands; 8 Department of Radiology, The Hague Medical Center, The Hague, the Netherlands; 9 Department of Radiology, Erasmus University Medical Center, Rotterdam, Netherlands

**Keywords:** CT perfusion, Ischemic core, Stroke, Thrombectomy, Alteplase

## Abstract

**Purpose:**

Intravenous alteplase (IVT) prior to endovascular treatment (EVT) is neither superior nor noninferior to EVT alone in acute ischemic stroke patients. We aim to assess whether the effect of IVT prior to EVT differs according to CT perfusion (CTP)–based imaging parameters.

**Methods:**

In this retrospective post hoc analysis, we included patients from the MR CLEAN-NO IV with available CTP data. CTP data were processed using syngo.via (version VB40). We performed multivariable logistic regression to obtain the effect size estimates (adjusted common odds ratio a[c]OR) on 90-day functional outcome (modified Rankin Scale [mRS]) and functional independence (mRS 0-2) for CTP parameters with two-way multiplicative interaction terms between IVT administration and the studied parameters.

**Results:**

In 227 patients, median CTP-estimated core volume was 13 (IQR 5–35) mL. The treatment effect of IVT prior to EVT on outcome was not altered by CTP-estimated ischemic core volume, penumbral volume, mismatch ratio, and presence of a target mismatch profile. None of the CTP parameters was significantly associated with functional outcome after adjusting for confounders.

**Conclusion:**

In directly admitted patients with limited CTP-estimated ischemic core volumes who presented within 4.5 h after symptom onset, CTP parameters did not statistically significantly alter the treatment effect of IVT prior to EVT. Further studies are needed to confirm these results in patients with larger core volumes and more unfavorable baseline perfusion profiles on CTP imaging.

**Supplementary Information:**

The online version contains supplementary material available at 10.1007/s00234-023-03139-4.

## Introduction

Six randomized trials recently compared the added value and risk of endovascular treatment (EVT) alone with intravenous thrombolysis (IVT) using alteplase prior to EVT in patients with acute ischemic stroke due to a large vessel occlusion in the anterior circulation [1–6]. The Chinese DIRECT-MT and DEVT trials showed non-inferiority of EVT alone whilst the other four trials (including The Multicenter Randomized Clinical Trial of Endovascular Treatment for Acute Ischemic Stroke in the Netherlands (MR CLEAN)–NO IV trial; ISRCTN80619088) neither showed superiority nor non-inferiority of EVT alone [1–6]. Therefore, the recently published guideline from the European Stroke Organisation (ESO) and European Society for Minimally Invasive Neurological Therapy (ESMINT) recommends IVT prior to EVT over EVT alone [7].

In general, patients with extensive hypoperfusion on baseline imaging are considered more suitable candidates for EVT alone — hypothesizing that patients with more extensive infarct at baseline have a higher risk of hemorrhagic transformation after IVT using alteplase [8, 9]. The extent of the baseline infarct is commonly estimated using CT perfusion (CTP).

The effect of IVT in patients who are eligible for EVT may be impacted by the baseline infarct volume [9]. CTP enables estimating the baseline ischemic core volume and could therefore potentially identify patients with reduced benefit from IVT, e.g., due to an increased risk of hemorrhagic transformation after IVT [9–11]. Thus far, one study has assessed the modification of IVT treatment effect prior to EVT by baseline infarct size, assessed with Alberta Stroke Program Early CT Score (ASPECTS), and found that baseline infarct size did not modify the treatment effect of IVT prior to EVT [12]. Additionally, subgroup analyses of the Direct Endovascular Thrombectomy vs Combined IVT and Endovascular Thrombectomy for Patients With Acute Large Vessel Occlusion in the Anterior Circulation (DEVT) and The Direct Mechanical Thrombectomy in Acute LVO Stroke also found no treatment effect heterogeneity based on ASPECTS dichotomization (i.e., ASPECTS <8 vs. ASPECTS 8–10). However, none of these prior analyses did include CTP parameters [2, 6].

In this post hoc analysis of the MR CLEAN-NO IV trial, we determine whether the effect of IVT prior to EVT on functional outcome differs according to CTP-estimated ischemic core volume, penumbral volume, mismatch ratio, and presence of a target mismatch (TMM) profile in EVT-eligible patients who directly presented to an EVT-capable center within 4.5 h after symptom onset.

## Methods

### Patient selection

The MR CLEAN-NO IV trial was an international, multicenter, prospective randomized open-label clinical trial which randomized patients who directly presented at an EVT-capable center — and were eligible for both IVT and EVT — to either IVT (alteplase 0.9 mg/kg) prior to EVT or EVT alone between January 2018 and October 2020. The study methods and patient eligibility criteria were published previously [13]. In this post hoc analysis, we included all patients with available baseline CTP results.

### Baseline imaging acquisition, post-processing, and quality assessment

Baseline NCCT and CTA data were scored by an independent core lab of neuroradiologists [13]. Observers were blinded to all clinical information except for occlusion side. CTP images were acquired according to local acquisition protocols per site. CTP data were centrally post-processed by an independent core laboratory using *syngo.via* (version VB40, Siemens Healthineers, Forchheim, Germany). The “ischemic core” was estimated as CBV <1.2 mL/100mL. Critically hypoperfused — yet not ischemic — tissue was defined as CBF <27 mL/100mL/min. A default smoothing filter was applied [14]. The penumbral volume was calculated as critically hypoperfused volume minus the ischemic core volume. The mismatch ratio was defined as the critically hypoperfused volume divided by the ischemic core volume. Presence of a TMM profile was defined as ischemic core volume <70 mL, mismatch ratio >1.8, and penumbral volume ≥15 mL [15]. Visual quality assessment of the CTP results was performed by two experienced neuroradiologists (>10 and >15 years of experience).

### Outcomes

The primary outcome was functional outcome — scored on the ordinal modified Rankin Scale (mRS) — at 90 days. The secondary outcome was functional independence (i.e., mRS 0–2) at 90 days. Occurrence of symptomatic intracerebral hemorrhage (sICH) scored according to the Heidelberg Bleeding Classification was the safety end point [16].

### Statistical analyses

We report the adjusted common odds ratio (a[c]OR) with 95% confidence intervals (95% CI) for a shift towards improved functional outcome on the mRS at 90 days. Results are reported per 10 mL (or per 10 percentage point [p.p.] for mismatch ratio) increase. We used ordinal and binary uni- and multivariable logistic regression models with and without two-way multiplicative interaction terms (between the studied parameters and IVT administration) to assess whether the treatment effect of IVT prior to EVT was modified by CTP-estimated ischemic core, penumbral volume, mismatch ratio, and presence of a TMM profile. Similarly, we aimed to analyze the relationship between functional outcome and the abovementioned CTP parameters. We adjusted the multivariable regression models for the following variables: age, pre-stroke mRS, onset-to-randomization time, and NIHSS score at baseline. We performed sensitivity analyses to assess the rates of reperfusion after EVT (eTICI score), follow-up lesion volume (FLV), and functional independence for subgroups stratified by presence of a TMM profile. Baseline clinical and imaging characteristics of both treatment arms were compared using Mann-Whitney *U* and *χ*^2^ tests. Five (2%) patients had missing variables. These patients were excluded from our analyses. We considered a two-sided *p*<0.05 as statistically significant. Statistical analyses were performed using RStudio (R Statistical Software, v2022.02.2, R: A language and environment for statistical computing, R Foundation for Statistical Computing, Vienna, Austria).

### Protocol approval and patient consent

The MR CLEAN-NO IV trial protocol was approved by national central ethical committees and by research boards at each participating center. The final versions of the trial protocol and statistical analysis plan are both available at www.NEJM.org. The MR CLEAN-NO IV trial was conducted in accordance with the revised Helsinki guidelines.

### Data availability statement

Data will be made available upon reasonable request to the principal investigators of the MR CLEAN-NO IV trial, from 18 months after publication of the main paper (November 11, 2021). A Data Sharing Statement is available at www.NEJM.org.

## Results

### Patient cohort and baseline characteristics

**Fig. 1:**
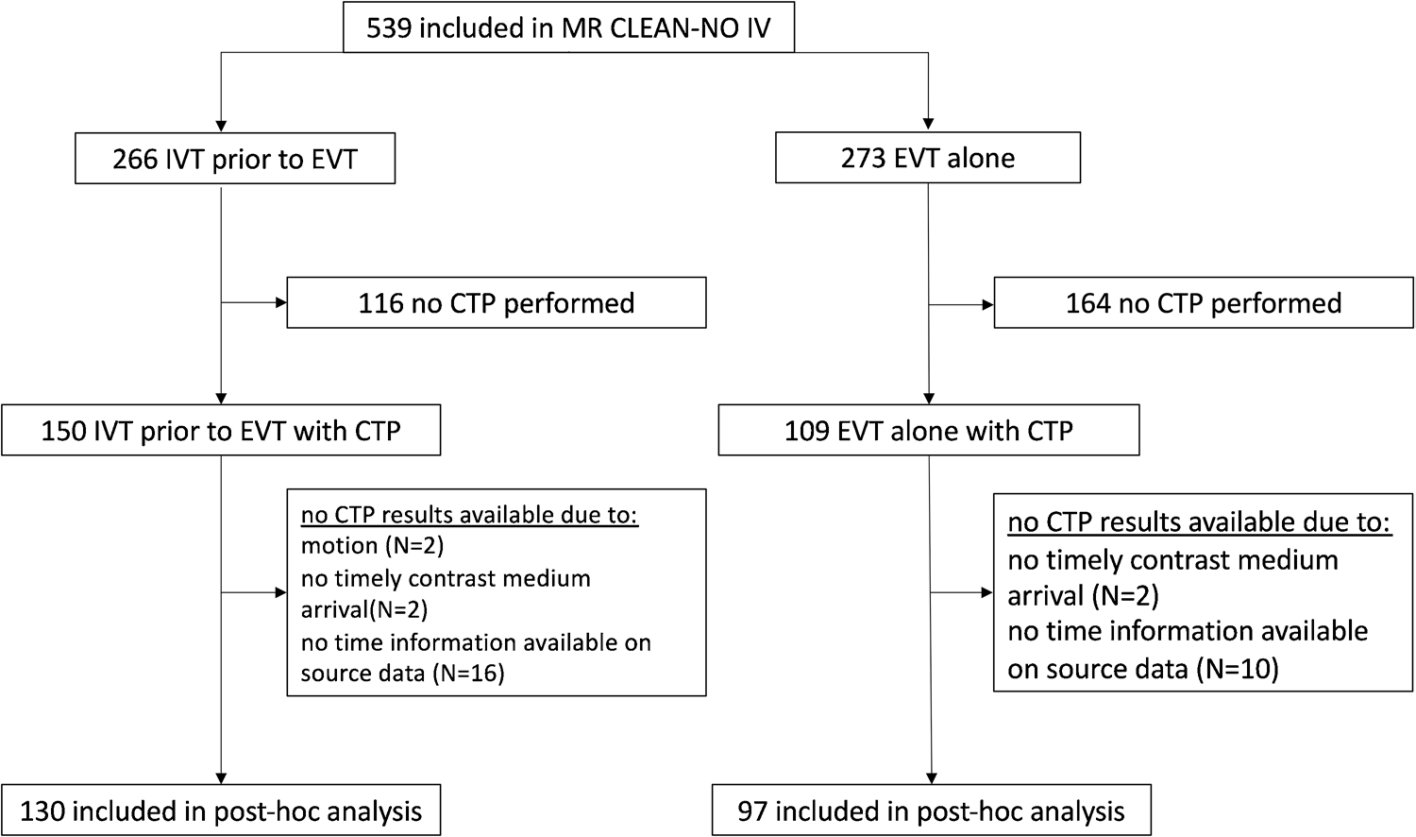
Flowchart of patient selection. CTP, CT perfusion; EVT, endovascular treatment; IVT, intravenous alteplase

A flowchart of patient selection is shown in Fig. 1. Of 539 patients included in the MR CLEAN-NO IV trial, 259 (48%) patients underwent CTP at admission. CTP results were available for 227/259 (88%) patients. Reasons not to perform CTP at baseline were mostly related to differences in local baseline imaging protocols. For example, some participating centers did not include CTP in the stroke imaging protocol during the inclusion period. Second not all centers in the Netherlands did perform CTP for patients presenting within 6 h after symptom onset at the time of inclusion. Other reasons include transfer from a primary stroke center without CTP-capabilities and local storage of CTP data. The decision to perform CTP was not affected by baseline characteristics such as, for example, baseline stroke severity or onset-to-imaging time. This is also reflected by the similarities in baseline characteristics with the overall MR CLEAN-NO IV population (Table 1). CTP data could not be processed for 32 patients for the following reasons: severe patient motion (*n*=2), no timely contrast medium arrival or incorrect timing CTP (*n*=6), and absence of time information on CTP source data (*n*=24). In the included patients, median CTP ischemic core volume was 13 (IQR 5–35) mL, median penumbral volume was 114 (IQR 78–149 mL), and median mismatch ratio was 9.4 (IQR 4.6–18.7). Median ASPECTS at baseline was 9 (IQR 8–10). CTP ischemic core volume was associated with ASPECTS (*p*<0.001) and collateral status (*p*<0.001). Patients with absent or poor collaterals (i.e., CTA collateral score 1–2) had larger CTP-estimated core volumes compared to patients with CTA collateral score 3–4 (34 vs. 8 mL [*p*<0.001]).

**Table 1:** Baseline and outcome characteristics of the MR CLEAN-NO IV post hoc analysis subgroup compared to the overall MR CLEAN-NO IV trial cohort*. ASPECTS*, Alberta Stroke Program Early CT Score; *CTA-CS*, Computed Tomography Angiography Collateral Score; *CTP*, computed tomography perfusion; *ICA*, internal carotid artery; *ICA-T*, internal carotid artery terminus; *IVT*, IV alteplase; *IQR*, interquartinfarct growth range; *mRS*, modified Rankin Score; *NIHSS*, National Institute of Health Stroke Scale; *sICH*, symptomatic intracerebral hemorrhage. If the [known in] number is not shown, the variable was known for all patients

Baseline characteristic	Overall MR CLEAN-NO IV CTP subgroup (*n*=227)	MR CLEAN-NO IV CTP subgroup — IVT prior to EVT (*n*=130)	MR CLEAN-NO IV CTP subgroup — EVT alone (*n*=97)	Overall MR CLEAN-NO IV population (*n*=539)
Age (year), median(IQR)	70 (61–78)	70 (61–78)	70 (61–78)	71 (62–79)
Female, *n* (%)	96 (42)	58 (45)	38 (39)	234 (43)
Baseline NIHSS, median (IQR)	16 (11–20)	16 (10–20)	16 (11–20)	16 (10–20)
Prior antiplatelet use, *n* (%)	83 (37)	51 (39)	32 (33)	190 (35)
Median systolic blood pressure (IQR), mmHg	146 (129–165)	146 (128–164)	147 (130–166)	150 (133–169)
IVT administered, *n* yes (%)	130 (57)	130 (100)	0 (0)	285 (53)
Onset-to-imaging time (min), median (IQR) [known in]	67 (52–89) [*n*=147]	67 (55–88)	69 (51–91)	67 (53–89) [*n*=170]
Onset-to-groin time (min), median (IQR) [known in]	132 (105–172) [*n*=214]	130 (105–161)	135 (107–180)	133 (105–180) [*n*=511]
Onset-to-needle time (min), median (IQR) [known in]	92 (76–131) [*n*=120]	92 (76–131)	NA	100 (75–157) [*n*=260]
*Imaging*				
Occlusion location on baseline CTA, *n* (%)				
*ICA*	2 (1)	0 (0)	2 (2)	4 (1)
*ICA-T*	50 (22)	23 (18)	27 (28)	114( 21)
*M1*	136 (60)	85 (65)	51 (53)	330 (61)
*M2*	37 (16)	21 (16)	16 (17)	85 (16)
*Other*	2 (1)	1 (1)	1 (1)	5 (1)
ASPECTS, median (IQR)	9 (8–10)	9 (8–10)	9 (8–10)	9 (8–10)
CTA-CS, *n* (%) [known in]	[*n*=220]	[*n*=124]	[*n*=96]	[*n*=526]
*0*	14 (6)	7 (5)	7 (7)	32 (6)
*1*	57 (26)	38 (29)	19 (20)	152 (29)
*2*	99 (45)	51 (39)	48 (50)	223 (42)
*3*	50 (23)	28 (22)	22 (23)	119 (23)
Ischemic core volume on CTP (mL), median (IQR)	13 (5–35)	15 (4–33)	12 (7–40)	13 (5–35) [*n*=227]
Penumbral volume on CTP (mL), median (IQR)	114 (78–149)	118 (78–150)	109 (81–145)	114 (78–149) [*n*=227]
Mismatch ratio (p.p.), median (IQR)	9.4 (4.6–18.7)	9.0 (4.7–20.1)	9.9 (4.3–18.1)	9.4 (4.6–18.7) [*n*=227]
Present target mismatch profile, *n* (%)	196 (86)	112 (86)	84 (87)	196 (86) [*n*=227]
eTICI, *n* (%) [known in]	[*n*=204]	[*n*=113]	[*n*=91]	[*n*=480]
*0*	15 (7)	10 (8)	5 (5)	36 (8)
*1*	4 (2)	3 (2)	1 (1)	6 (1)
*2a*	16 (8)	9 (7)	7 (7)	50 (10)
*2b*	52 (26)	26 (20)	26 (27)	109 (23)
*2c*	25 (12)	12 (9)	13 (13)	59 (12)
*3*	92 (45)	53 (41)	39 (40)	220 (46)
*Outcomes*				
Poor functional outcome (mRS 5–6), *n* (%)	54 (24)	28 (22)	26 (27)	153 (28)
Functional independence (mRS 0–2), *n* (%)	125 (55)	71 (55)	55 (57)	270 (50)
Mortality at 90 days, *n* (%)	34 (15)	18 (14)	16 (17)	98 (18)
sICH, *n* (%)	12 (5)	8 (6)	4 (4)	30 (5)

One hundred thirty (58%) patients received IVT prior to EVT. Any intracerebral hemorrhage occurred in 63 (28%) patients (27/97 [28%] patients with IVT prior to EVT vs. 36/130 [28%] patients who underwent EVT alone, *p*=0.5). Symptomatic intracerebral hemorrhage occurred in 12 (5.3%) patients and did not differ per treatment arm (*p=*0.5). Baseline characteristics per treatment arms (i.e., IVT prior to EVT vs. EVT alone) are summarized in Table 1.

### Association between CTP ischemic core volume and functional outcome

CTP-estimated ischemic core volume was inversely associated with improved functional outcome at 90 days in the baseline univariable ordinal regression analysis (cOR per 10 mL 0.81 [95% CI 0.75–0.87], *p*<0.001) and in ordinal regression with a multiplicative interaction term applied (i.e., CTP ischemic core volume × IVT administration) (cOR 0.73 [95% CI 0.54–0.95], *p*=0.02). After adjusting for confounders, this association was no longer statistically significant (acOR per 10 mL 0.80 [95% CI 0.60–1.04]). The treatment effect of IVT prior to EVT was not modified by CTP ischemic core volume at baseline (acOR per 10 mL 1.01 [95% CI 0.86–1.19]). In total, 125/227 (55%) patients with CTP results available achieved functional independence at 90 days. The proportion of patients who achieved functional independence did not differ per study arm (71/130 [55%] patients with IVT prior to EVT vs. 54/97 [56%] patients who underwent EVT alone, *p*=0.8). In univariable analysis, CTP ischemic core volume (cOR per 10 mL 0.80 [95% CI 0.71–0.88], *p*<0.001) was significantly associated with achieving functional independence at 90 days. After adjusting for confounders, this association was no longer statistically significant (acOR per 10 mL 0.89 [95% CI 0.60–1.29]). A detailed presentation of the univariable associations of clinical and imaging parameters (i.e., CTP parameters, ASPECTS, age, pre-stroke mRS, baseline NIHSS, and onset-to-randomization time) with improved functional outcome are given in Supplemental Table I and Supplemental Table II. Detailed results of the multivariable associations per CTP-based imaging biomarker with functional independence are provided in Supplemental Table III and Supplemental Table IV.

### Association between CTP penumbral volume, mismatch ratio, and functional outcome

**Fig. 2:**
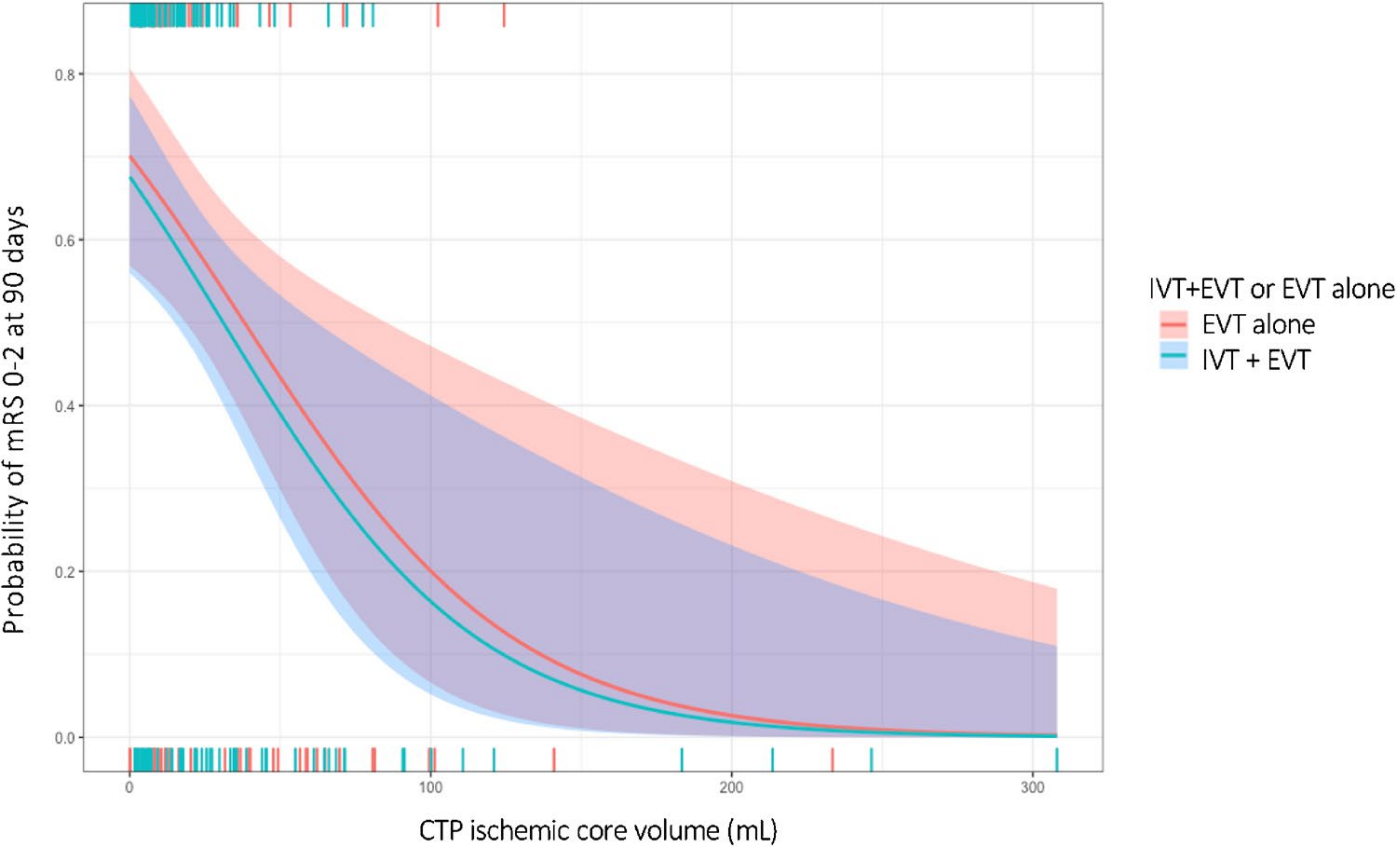
Probability of functional independence for CTP-estimated ischemic core volume adjusted for potential confounders. Associations are shown for patients who received IVT prior to EVT (blue) and who underwent EVT alone (red). The horizontal bars above and below the graph represent the CTP-estimated ischemic core volumes for patients who did achieve or did not achieve functional independence at 90 days, respectively. Each vertical line represents one patient. Patients with IVT prior to EVT are shown in blue and patients who underwent EVT alone are shown in red. CTP, computed tomography perfusion; EVT, endovascular treatment; IVT, intravenous alteplase; mRS, modified Rankin Scale score

Penumbral volume was not associated with improved functional outcome at 90 days in uni- (cOR per 10 mL 0.98 [95% CI 0.94–1.03]) or multivariable analyses (acOR per 10 mL 1.10 [95% CI 0.93–1.30]). We neither observed a statistically significant association between penumbral volume and functional independence at 90 days in uni- (cOR per 10 mL 0.97 [95% CI 0.92–1.03]) or multivariable analyses (acOR per 10 mL 1.20 [95% CI 0.96-1.50]). Mismatch ratio was associated with improved functional outcome at 90 days (cOR per 10 p.p. 1.17 [95% CI 1.06-1.31], *p*<0.01). We did not observe this association in multivariable analysis (acOR per 10 p.p. 1.07 [95% CI 0.71–1.66]). In addition, mismatch ratio was associated with functional independence at 90 days in univariable analysis (cOR per 10 p.p. 1.17 [95% CI 1.02–1.37], *p*=0.04), but not after adjusting for confounders (1.25 per 10 p.p. [95% CI 0.68–2.58]). The treatment effect of IVT prior to EVT was not modified by either the penumbral volume or mismatch ratio at baseline. Figure 2 shows the adjusted correlations between CTP-estimated ischemic core volume and functional independence at 90 days per study arm.

### Association between target mismatch (TMM) profile and functional outcome

**Fig. 3:**
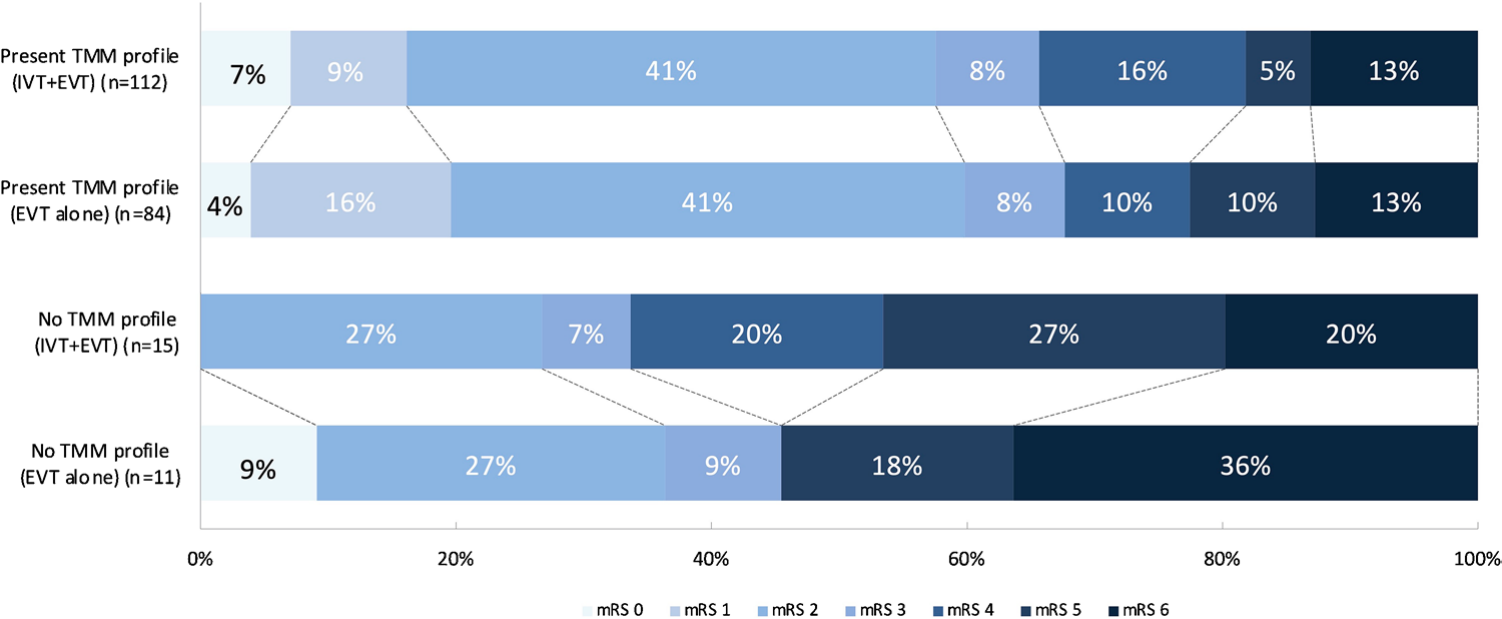
Distribution of scores on the Modified Rankin Scale score at 90 days in the MR CLEAN-NO IV CTP subgroup for patients with (*n*=196) and without (*n*=26) a target mismatch (TMM) profile on CTP imaging who underwent IVT and EVT vs. EVT alone. CTP, computed tomography perfusion; mRS, modified Rankin Scale score at 90 days

One hundred ninety-six (86%) patients had a TMM profile. Patients with a TMM profile equally often received IVT prior to EVT compared to patients without a TMM profile (57% vs. 58%). One hundred fourteen (58%) patients with a TMM profile achieved functional independence at 90 days vs. 8 (31%) of patients without a TMM profile (*p*<0.01). Presence of a TMM profile was associated with improved functional outcome in univariable analysis (cOR 3.15 [95% CI 1.51–6.57], *p*<0.002), but not in multivariable analysis (acOR 1.64 [95% CI 0.13–21.2]). Presence of a TMM profile was statistically significantly associated with functional independence at 90 days in univariable analysis (cOR 3.13 [95% CI 1.34–7.94], *p*=0.01), but not after adjusting for confounders (acOR 1.18 [95% CI 0.04–35.33], *p*=0.9). Forty (20%) patients with a TMM profile showed poor outcome (i.e., mRS 5–6) at 90 days compared to thirteen (50%) of patients without a TMM profile. The distributions of the mRS scores at 90 days for patients with and without a TMM profile stratified by treatment allocation is shown in Fig. 3.

### Association between CTP parameters and intracerebral hemorrhage and sICH

None of the CTP parameters or clinical parameters was statistically significantly associated with the occurrence of sICH or any intracerebral hemorrhage. Detailed results of the univariable associations of clinical and imaging parameters with sICH are given in Supplemental Table V.

## Discussion

This post hoc analysis of the MR CLEAN-NO IV trial showed that the treatment effect of IVT prior to EVT in the hyperacute — 0–4.5 h — time window was not statistically significantly modified by CTP ischemic core volume, penumbral volume, mismatch ratio, or presence of a TMM profile. In addition, our results did not show an association between CTP parameters and the occurrence of sICH. However, our study sample was limited, and most patients had relatively limited perfusion deficits on CTP imaging with substantial collateral flow on CTA. If replicated in a pooled analysis with more patients with more extensive perfusion deficits, our findings provide preliminary evidence that CTP parameters may not be able identify patients who are less likely to benefit from IVT prior to EVT.

Similar to our findings, a recent post hoc analysis of the DIRECT-MT trial showed that the baseline infarct size — estimated using ASPECTS — did not modify the treatment effect of IV alteplase prior to EVT [12]. This is in line with subgroup analyses from the Direct Endovascular Thrombectomy vs Combined IVT and Endovascular Thrombectomy for Patients With Acute Large Vessel Occlusion in the Anterior Circulation (DEVT) and The Direct Mechanical Thrombectomy in Acute LVO Stroke (SKIP) trials which did not show treatment effect heterogeneity based on ASPECTS dichotomized into ASPECTS <8 vs. ASPECTS 8–10 [2, 6]. Furthermore, in the DIRECT-MT trial, patients with extensive baseline infarction (i.e., ASPECTS 0–4) were highly unlikely to achieve functional independence at 90 days regardless of IVT administration prior to EVT [12]. More specifically, only 3/25 (12%) patients with ASPECTS 0–4 achieved functional independence in the EVT alone arm compared to 5/31 (16%) of patients with ASPECTS 0–4 in the IVT prior to EVT arm. Due to the low number of patients with large CTP-estimated ischemic core volumes in the MR CLEAN-NO IV trial, our results might not be applicable to patients with extensive perfusion deficits on baseline CTP.

Our findings are in contrast with a previous observational study from the MR CLEAN Registry, which collected data from stroke patients in the same healthcare system as the MR CLEAN-NO IV trial and found that CTP ischemic core volume was associated with functional outcome at 90 days [17]. Although the rate of IVT prior to EVT was higher in the MR CLEAN Registry (i.e., 72%) and patients who present later in the 0–6 h time window may have more established infarcts, it is most likely that the contradictory conclusions can be explained by the limited sample size in both studies in addition to the fact that the accuracy of CTP may be different for patients who present in the hyperacute (i.e., 0–4.5 h) vs. the early (0–6 h) time window [18].

Several limitations of our analysis should be noted. First, the median estimated ischemic core volumes were relatively small (i.e., 13 mL). Although this is similar to the median CTP-estimated ischemic core volumes from a cohort of patients treated with EVT in daily clinical practice in the Netherlands, our results might not be applicable to patients with more extensive perfusion deficits [17]. Second, we were not able to include the hypoperfusion intensity ratio (HIR) as this is currently not provided by the used CTP analysis software. The HIR is a CTP imaging biomarker which could be used as a surrogate marker of collateral circulation and has been shown to be associated with functional outcome in patients with acute ischemic stroke [19].

Third, CTP was performed according to local acquisition protocols and therefore not routinely acquired in every admitted suspected stroke patient. As differences in acquisition protocols may influence the CTP results [20], this could have affected our results. However, all CTP data were centrally processed using a single software package using a previously validated procedure. Fourth, only 259/539 (48%) patients included in the MR CLEAN-NO IV trial underwent baseline CTP imaging. Together with the fact that 32 CTP datasets could not be processed, this resulted in a relatively limited sample size. Finally, the MR CLEAN-NO IV trial only included directly admitted patients who could be treated within 4.5 h after stroke onset. Therefore, our results are not generalizable to the extended — 0–6 h and 0–9 h — time windows for IVT administration in acute ischemic stroke patients.

## Conclusions

In directly admitted patients with limited CTP-estimated ischemic core volumes who presented within 4.5 h after symptom onset, CTP parameters did not statistically significantly alter the treatment effect of IVT prior to EVT. Further studies are needed to confirm these results in patients with larger core volumes and more unfavorable baseline perfusion profiles on CTP imaging.

## Supplementary Information

Below is the link to the electronic supplementary material.

Supplementary file1 (DOCX 40 KB)

## Abbreviations

EVT: Endovascular treatment
FLV: Follow-up lesion volume
IVT: Intravenous thrombolysis
TMM: Target mismatch
sICH: Symptomatic intracranial hemorrhage
