# Supplementary material for: Association between computed tomography perfusion and the effect of intravenous alteplase prior to endovascular treatment in acute ischemic stroke
Source: Neuroradiology. 2023 Mar 8;65(6):1053–61. doi: 10.1007/s00234-023-03139-4 (PMC10169898; doi:10.1007/s00234-023-03139-4)
Supplement: Supplementary file 1 — Supplementary file1 (DOCX 40 KB) [file 234_2023_3139_MOESM1_ESM.docx]

**SUPPLEMENTAL MATERIAL**

| **Supplemental Table I. Univariable analysis of improved functional outcome (mRS) at 90 days.** ASPECTS, Alberta Stroke Program Early CT Score; IVT, intravenous alteplase; mRS, modified Rankin Scale; NIHSS, National Institutes of Health Stroke Scale; OR, Odds Ratio. | | |
| --- | --- | --- |
| **Variables** | **OR (95% CI)** | ***P* value** |
| **CTP ischemic core volume (per 10 mL)** | **0.81 (0.75-0.87)** | **<0.001** |
| CTP penumbral volume (per 10 mL) | 0.98 (0.94-1.03) | 0.49 |
| **CTP mismatch ratio (per 10 percentage point)** | **1.17 (1.06-1.31)** | **<0.01** |
| **Present CTP target mismatch (TMM) profile** | **3.15 (1.51-6.57)** | **0.002** |
| **ASPECTS** | **1.17 (1.00-1.37)** | **0.05** |
| **Age (per year)** | **0.96 (0.94-0.98)** | **<0.001** |
| **Pre-stroke mRS** | **0.62 (0.46-0.82)** | **0.001** |
| **NIHSS at baseline (per point)** | **0.88 (0.85-0.92)** | **<0.001** |
| **Onset-to-randomization time (per 10 min)** | **0.95 (0.91-0.99)** | **0.01** |
| IVT administration | 0.99 (0.61-1.59) | 0.96 |

| **Supplemental Table II. Univariable analysis of functional independence (mRS 0-2).** ASPECTS, Alberta Stroke Program Early CT Score; CI, confidence interval; IVT, intravenous alteplase; mRS, modified Rankin Scale; NIHSS, National Institutes of Health Stroke Scale; OR, Odds Ratio. | | |
| --- | --- | --- |
| **Variables** | **OR (95% CI)** | ***P* value** |
| **CTP ischemic core volume (per 10 mL**) | **0.80 (0.71-0.88)** | **<0.001** |
| CTP penumbral volume (per 10 mL) | 0.97 (0.92-1.03) | 0.34 |
| **CTP mismatch ratio (per 10 percentage point)** | **1.17 (1.02-1.37)** | **0.04** |
| **Present CTP target mismatch (TMM) profile** | **3.13 (1.34-7.94)** | **0.01** |
| ASPECTS | 1.12 (0.95-1.34) | 0.18 |
| **Age (per year)** | **0.95 (0.93-0.97)** | **<0.001** |
| **Pre-stroke mRS** | **0.57 (0.39-0.81)** | **0.002** |
| **NIHSS at baseline (per point)** | **0.88 (0.84-0.93)** | **<0.001** |
| **Onset-to-randomization time (per 10 min)** | **0.93 (0.89-0.98)** | **0.01** |
| IVT administration | 0.94 (0.55-1.59) | 0.81 |

| **Supplemental Table III. Multivariable analysis of improved functional outcome (mRS) at 90 days.** ASPECTS, Alberta Stroke Program Early CT Score; intravenous alteplase; mRS, modified Rankin Scale; NIHSS, National Institutes of Health Stroke Scale; aOR , adjusted Odds Ratio. | | | |
| --- | --- | --- | --- |
| **Variables** | | **aOR(95% CI)** | ***P* value** |
| CTP ischemic core volume (per 10 mL) | | 0.80 (0.60-1.04) | 0.10 |
|  | **Age (per year)** | **0.97 (0.95-0.99)** | **0.002** |
|  | **Pre-stroke mRS** | **0.59 (0.41-0.80)** | **<0.001** |
|  | **Onset-to-randomization time  (per 10 min)** | **0.92 (0.88-0.97)** | **<0.001** |
|  | **NIHSS at baseline (per point)** | **0.90 (0.86-0.94)** | **<0.001** |
|  | IVT administration | 1.24 (0.65-2.37) | 0.52 |
|  | CTP ischemic core volume × IVT administration | 1.01 (0.86-1.19) | 0.89 |
| CTP penumbral volume (per 10 mL) | | 1.10 (0.93-1.30) | 0.28 |
|  | **Age (per year)** | **0.97 (0.95-0.98)** | **0.001** |
|  | **Pre-stroke mRS** | **0.72 (0.52-0.99)** | **0.049** |
|  | **Onset-to-randomization time  (per 10 min)** | **0.93 (0.89-0.97)** | **0.001** |
|  | **NIHSS at baseline (per point)** | **0.87 (0.83-0.91)** | **<0.001** |
|  | IVT administration | 2.05 (0.61-7.02) | 0.25 |
|  | CTP penumbral core volume × IVT administration | 0.95 (0.86-1.05) | 0.31 |
| CTP mismatch ratio (per 10 percent points) | | 1.07 (0.71-1.66) | 0.75 |
|  | **Age (per year)** | **0.97 (0.95-0.99)** | **0.001** |
|  | **Pre-stroke mRS** | **0.65 (0.47-0.91)** | **0.013** |
|  | **Onset-to-randomization time  (per 10 min)** | **0.92 (0.88-0.97)** | **0.001** |
|  | **NIHSS at baseline (per point)** | **0.88 (0.84-0.92)** | **<0.001** |
|  | **IVT administration** | **1.02 (0.54-1.92)** | **0.954** |
|  | CTP mismatch ratio × IVT administration | 1.05 (0.82-1.34) | 0.69 |
| Present CTP target mismatch (TMM) profile | | 1.64 (0.13-21.2) | 0.7 |
|  | **Age (per year)** | **0.97(0.95-0.98)** | **<0.001** |
|  | **Pre-stroke mRS** | **0.65 (0.47-0.90)** | **0.01** |
|  | **Onset-to-randomization time  (per 10 min)** | **0.93 (0.88-0.97)** | **0.001** |
|  | **NIHSS at baseline (per point)** | **0.87 (0.84-0.91)** | **<0.001** |
|  | IVT administration | 0.77 (0.19-3.16) | 0.7 |
|  | CTP target mismatch profile × IVT administration | 1.59 (0.35-7.14) | 0.5 |
| ASPECTS | | 1.23 (0.71-2.21) | 0.47 |
|  | **Age (per year)** | **0.96 (0.94-0.98)** | **<0.001** |
|  | **Pre-stroke mRS** | **0.72 (0.52-0.99)** | **0.04** |
|  | **Onset-to-randomization time  (per 10 min)** | **0.93 (0.89-0.98)** | **0.003** |
|  | **NIHSS at baseline (per point)** | **0.88 (0.84-0.92)** | **<0.001** |
|  | IVT administration | 1.57 (0.09-31.3) | 0.76 |
|  | ASPECTS × IVT administration | 0.97 (0.69-1.34) | 0.85 |

| **Supplemental Table IV. Multivariable analysis of functional independence (mRS 0-2).** ASPECTS, Alberta Stroke Program Early CT Score; IVT, intravenous alteplase; mRS, modified Rankin Scale; NIHSS, National Institutes of Health Stroke Scale; aOR , adjusted Odds Ratio. | | | |
| --- | --- | --- | --- |
| **Variables** | | **aOR(95% CI)** | ***P* value** |
| CTP ischemic core volume (per 10 mL) | | 0.89 (0.60-1.29) | 0.55 |
|  | **Age (per year)** | **0.95 (0.92-0.97)** | **<0.001** |
|  | **Pre-stroke mRS** | **0.57 (0.37-0.87)** | **0.01** |
|  | **Onset-to-randomization time  (per 10 min)** | **0.90 (0.84-0.96)** | **0.001** |
|  | **NIHSS at baseline (per point)** | **0.90 (0.84-0.95)** | **<0.001** |
|  | IVT administration | 1.02 (0.42-2.46) | 0.96 |
|  | CTP ischemic core volume × IVT administration | 0.93 (0.72-1.18) | 0.55 |
| CTP penumbral volume (per 10 mL) | | 1.20 (0.96-1.50) | 0.1 |
|  | **Age (per year)** | **0.95 (0.92-0.97)** | **<0.001** |
|  | **Pre-stroke mRS** | **0.66 (0.42-0.99)** | **0.05** |
|  | **Onset-to-randomization time  (per 10 min)** | **0.91 (0.85-0.96)** | **0.002** |
|  | **NIHSS at baseline (per point)** | **0.86 (0.81-0.91)** | **<0.001** |
|  | IVT administration | 3.42 (0.69-17.55) | 0.14 |
|  | CTP penumbral core volume × IVT administration | 0.89 (0.78-1.01) | 0.07 |
| CTP mismatch ratio (per 10 percentage points) | | 1.25 (0.68-2.58) | 0.51 |
|  | **Age (per year)** | **0.95 (0.92-0.98)** | **<0.001** |
|  | **Pre-stroke mRS** | **0.63 (0.40-0.95)** | **0.033** |
|  | **Onset-to-randomization time  (per 10 min)** | **0.92 (0.88-0.97)** | **0.002** |
|  | **NIHSS at baseline (per point)** | **0.87 (0.82-0.92)** | **<0.001** |
|  | **IVT administration** | **0.86 (0.38-1.98)** | **0.72** |
|  | CTP mismatch ratio × IVT administration | 0.97 (0.65-1.39) | 0.86 |
| Present CTP target mismatch (TMM) profile | | 1.18 (0.04-35.2) | 0.92 |
|  | **Age (per year)** | **0.95 (0.92-0.98)** | **<0.001** |
|  | **Pre-stroke mRS** | **0.62 (0.40-0.94)** | **0.03** |
|  | **Onset-to-randomization time  (per 10 min)** | **0.91 (0.85-0.96)** | **0.002** |
|  | **NIHSS at baseline (per point)** | **0.87 (0.82-0.92)** | **<0.001** |
|  | IVT administration | 0.48 (0.07-3.20) | 0.4 |
|  | CTP target mismatch profile × IVT administration | 1.95 (0.35-7.14) | 0.5 |
| ASPECTS | | 1.00 (0.49-2.15) | 0.99 |
|  | **Age (per year)** | **0.95 (0.92-0.97)** | **<0.001** |
|  | **Pre-stroke mRS** | **0.65 (0.42-0.98)** | **0.04** |
|  | **Onset-to-randomization time  (per 10 min)** | **0.91 (0.85-0.96)** | **0.002** |
|  | **NIHSS at baseline (per point)** | **0.87 (0.82-0.92)** | **<0.001** |
|  | IVT administration | 10.46 (0.01-24.01) | 0.69 |
|  | ASPECTS × IVT administration | 1.08 (0.69-1.66) | 0.72 |

| **Supplemental Table V. Univariable analysis of symptomatic intracerebral hemorrhage (sICH).** ASPECTS, Alberta Stroke Program Early CT Score; CI, confidence interval; IVT, intravenous alteplase; mRS, modified Rankin Scale; NIHSS, National Institutes of Health Stroke Scale; OR, Odds Ratio. | | |
| --- | --- | --- |
| **Variables** | **OR (95% CI)** | ***P* value** |
| CTP ischemic core volume (per 10 mL) | 0.94 (0.73-1.09) | 0.55 |
| CTP penumbral volume (per 10 mL) | 0.98 (0.87-1.10) | 0.75 |
| CTP mismatch ratio (per 10 percentage point) | 1.00 (0.71-1.21) | 1.0 |
| Present CTP target mismatch (TMM) profile | 1.49 (0.27-27.74) | 0.71 |
| ASPECTS | 1.08 (0.76-1.74) | 0.71 |
| Age (per year) | 1.03 (0.98-1.08) | 0.26 |
| Pre-stroke mRS | 1.67 (0.89-2.93) | 0.09 |
| NIHSS at baseline (per point) | 1.01 (0.92-1.11) | 0.86 |
| Onset-to-randomization time (per 10 min) | 1.02 (0.91-1.13) | 0.65 |
| IVT administration | 1.52 (0.47-5.85) | 0.50 |
